# Assessing HER2 amplification by IHC, FISH, and real-time polymerase chain reaction analysis (real-time PCR) following LCM in formalin-fixed paraffin embedded tissue from 40 women with ovarian cancer

**DOI:** 10.1111/j.1600-0463.2012.02929.x

**Published:** 2012-06-26

**Authors:** Thore Hillig, Jørgen Thode, Marie F Breinholt, Maria-Benedicte Franzmann, Carsten Pedersen, Flemming Lund, Henrik Mygind, György Sölétormos, Martin Rudnicki

**Affiliations:** 1 Department of Clinical Biochemistry, Hillerød Hospital University of CopenhagenHillerød; 2 Department of Pathology, Roskilde Hospital University of CopenhagenRoskilde, Zealand Region; 3 Department of Pathology, Hospital SouthZealand Region; 4 Department of Gynecology, Roskilde Hospital University of CopenhagenZealand Region, Denmark

**Keywords:** HER2, laser capture microdissection, fluorescence in situ hybridization, ovarian cancer, diagnosis

## Abstract

We compare HER2 receptor amplification analysis by immunohistochemistry (IHC), fluorescence in situ hybridization (FISH), and real-time polymerase chain reaction (real-time PCR) DNA copy-number assay following laser capture microdissection (LCM) in formalin-fixed paraffin embedded tissue from 40 women with verified ovarian cancer. We speculate that LCM should result in a more accurate assessment of HER2 amplification in our real-time PCR assay compared with IHC and FISH. HER2 overexpression measured by IHC, FISH, or real-time PCR was found in 5.0%, 5.0%, and 22.5%, respectively. HER2 negative results measured by IHC, FISH, or real-time PCR were found in 95%, 92.5%, and 60.0%, respectively. Analysis failed for IHC, FISH, or real-time PCR in 0%, 2.5%, or 17.5% of cases. Concordance between IHC and FISH, IHC and real-time PCR, or FISH and real-time PCR were 89.7%, 72.7%, or 78.1%, respectively. Only few ovarian cancer patients were HER2 overexpressed measured by IHC or FISH and thus could be eligible for antibody-based therapy with trastuzumab (Herceptin). Interestingly, we find an increased number of HER2 positive patients by real-time PCR analysis on microdissected cancer cells, suggesting a number of HER2 positive patients not detected by current methods. Thus, the concept of quantitative measurement of HER2 on microdissected cancer cells should be explored further.

The HER2 oncogene encodes a transmembrane tyrosine kinase (Human Epidermal growth factor Receptor type 2) located on chromosome 17q21. Increased number (amplification) of this gene induces increased number of membrane receptors (overexpression). HER2 protein is one of four transmembrane receptor tyrosine kinases that are involved in intracellular signaling pathways that regulate cell growth and differentiation (1, 2). HER2 amplification is associated with accelerated disease progression and poor prognosis in malignancies afflicting women, e.g., in breast cancer and endometrial carcinoma (3–5). Amplification of the HER2/neu gene has been identified in 15–30% of breast cancer and endometrial cancer (6–8). The role of HER2 in ovarian cancer initiation and progression is less clearly known [9], and treatment with HER2 antagonists in ovarian cancer has thus far been disappointing (10, 11). Currently, the best method available for HER2 analysis recommended by the American Society of Clinical Oncology/College of American Pathologists (ASCO/CAP) guidelines is fluorescence in situ hybridization (FISH), which is generally viewed as superior to immunohistochemical (IHC) analysis and therefore used in both clinic and many research studies to evaluate IHC results, which show borderline reaction, e.g., IHC 2+ [12]. An analysis of HER2 needs to have a very high diagnostic sensitivity and specificity giving the patient with a severe disease the correct diagnosis and thus the correct treatment. Also overall health-cost calls for improved laboratory diagnostics as treatment with HER2 blocking agents is expensive.

The incidence of HER2 receptor amplification has been investigated in ovarian cancer with reported overexpression ranging between 5% and 27% (3–11). The correct diagnosis of HER2 amplified ovarian cancer patients is essential, and the methods used could have an impact on treatment decisions. Occasional reports of remission following trastuzumab therapy in HER2 negative ovarian cancer have raised the question whether the current methods for testing HER2 are sufficient and the use of molecular biology in clinical diagnostics is increasing [13]. However, no study has investigated HER2 amplification in ovarian cancer on a molecular biology basis by HER2 DNA gene quantification by real-time polymerase chain reaction (real-time PCR) in ovarian cancer. A complication in using real-time PCR and FISH from whole formalin prepared tissue is the presence of non-cancerous cells which can comprise 5–95% of a biopsy sample [12]. Thus, to have a pure cancer sample, it is necessary to capture the cancer cells by laser capture microdissection (LCM) and perform real-time PCR analysis on the cancer cells with minimal contamination with stromal cells [14].

We report results for HER2 amplification by IHC, FISH, and real-time PCR with a DNA copy-number assay on LCM cancer cells, in formalin-fixed paraffin embedded tissue from 40 women with ovarian cancer.

## Materials and Methods

### Materials

Patients: 40 women who underwent surgery for epithelial ovarian cancer during the years 1998–2003. Samples from patients were included consecutively during this period (Table 1).

**Table 1:** Summary of HER2 amplification analysis based on histology

Tumor type	No total	IHC neg−/pos+	FISH neg−/pos+	Real-time PCR neg−/pos+
Clear cell	4	4/0	4/0	3/0^1^
Endometrioid	15	15/0	13/1^1^	10/2^1^
Mucinous	5	5/0	5/0	4/1
Serous	12	12/0	11/1	5/4^1^
Miscellaneous^2^	4	4/0	4/0	2/2

HER2, human epidermal growth factor receptor type 2.

1 Samples not determined due to failure of analysis are not included in this table.

2 Miscellaneous consists of clear cell + serous, clear cell + endometrioid, and serous + endometrioid.

The inclusion criteria for women with ovarian cancer were epithelial ovarian cancer, age over 18, no additional present, or previous malignant conditions. Approval of the protocol was obtained from the local Danish Ethics Committee.

### Tissue preparation

From the tissue block containing carcinoma, four consecutive sections (2–4 microns) were made. First slide stained routinely with hematoxylin and eosin (HE), second slide IHC for HER2, third slide used for FISH analysis, and the fourth slide used for quantitative PCR analysis of HER2 gene amplification using LCM. Sections were mounted on electrostatically treated slides (Superfrost Plus®, Hounisen, Aarhus, Denmark) and heated to 65 °C for 25 min. Sections for LCM were mounted on polyethylene naphtalate membrane slides. The slides were then used directly for LCM using the HE-stained sections as guidance.

### IHC and FISH analysis

IHC was performed using the Leica Oracle HER2 Bond IHC system [Leica Microsystems GmbH, Wetzlar, Germany [15]]. The ZYTOVISION Zytolight® SPEC HER2/CEN 17 dual color probe assay (ZytoVision GmbH, Bremerhaven, Germany) was used to determine FISH amplification of the HER2 coding gene according to manufacturer's instructions. After proteolytic pretreatment, a ready to use FISH-probemix was applied to the slides. The probemix covers an approximately 600-kb large region including the HER2 gene on chromosome 17, and the centromeric region of chromosome 17 (CEN-17). Using a fluorescence microscope (Zeiss Axio Imager.Z1; Carl Zeiss AG, Feldbach, Switzerland), tumor cells are identified, the number of HER2 and CEN-17 signals counted to a total of 60 gene signals, and the HER2/CEN-17 ratio calculated. A sample with a HER2/CEN-17 ≥2.00 is considered HER2 gene amplified. A ratio of 1.8–2.0 is considered borderline and the signal recounted by a new investigator.

### Microdissection and real-time PCR

**Fig. 1:**
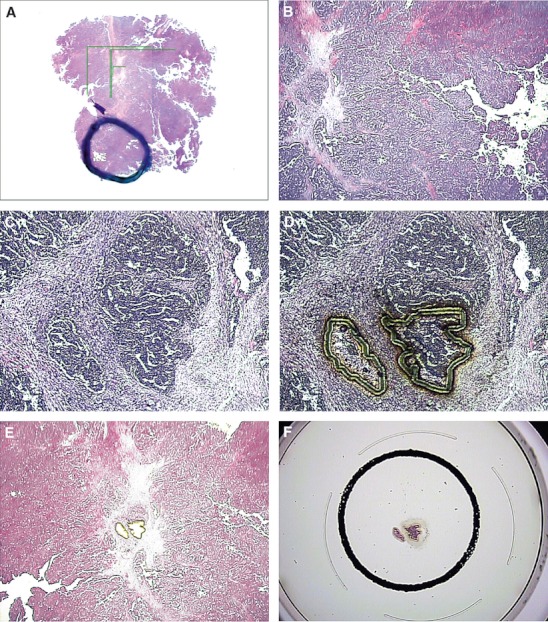
Microdissection of HE-stained ovarian cancer tissue slide. (A) overview of tissue slide, with ring marking area used for microdissection. (B) 20× magnified region of interest. (C) 100× region of interest with two cancer cell islets. (D) Laser cutting of region of interest. (E) 20× region of interest after laser capture microdissection. (F) Laser capture microdissected cells on cap, ready for subsequent analysis (real-time PCR).

Pretreated sections were inspected and microdissected (Fig. 1) by an Arcturus® LCM microscope (Life Technologies Corporation, Carlsbad, CA, USA). Approximately 600 cancer cells were microdissected from each slide onto a CapSure® LCM cap. DNA from LCM CapSure® caps were extracted by QIAamp® DNA Micro Kit (Qiagen GmbH, Hilden, Germany) according to manufacturer's instructions. Real-time PCR was performed on an ABI7500 thermocycler with 7500 software v2.03 (Life Technologies Corporation) by a slightly modified previously described method [16]. Briefly PCR reactions were performed in quadruplicate with a 15 μL final reaction volume consisting of 4 μL DNA, 1×TaqMan® Universal PCR Master, No AmpErase® UNG, 500 μM of HER2 primers (FW ATCTGCCTGACATCCACG, RV GCAATCTGCATACACCAGTTC), 700 μM of gastrin primers (FW TCTGAAGCTTCTTGGAAGCC, RV CCAGCTGCCTTCGATGA) and 250 nM of TaqMan® MGB probes (HER2 FAM-AGCTTATGCCCTATGGCT-MGB, gastrin VIC-AGATGCACCCTTAGGTACA-MGB) with all PCR reagents from Applied Biosystems (Life Technologies Corporation). Standard PCR conditions were used.

Modifications from original assay comprise original 20 μL final reaction volume replaced by 15 μL final reaction volume. Original lightcycler probes (HER2: GCCTCTTAGACCATGTCCGGGAAA-fluorescein and LC-Red 705-CGCGGACGCCTGGGCTC-phosphate, Gastrin: AGGGACCTGGAGCTACCCT-fluorescein and LC-Red 640-CTGGAGCAGCAGGGCCCAG-phosphate) are replaced by Taqman probes (see above). The PCR conditions were modified from 12 min at 95 °C of activation, gene amplification was performed over 55 cycles. Each of the cycles consisted of denaturation for 10 s at 95 °C, annealing for 10 s at 58 °C, and extension for 10 s at 72 °C. The fluorescence signals were measured after each primer-annealing step (58 °C), to standard PCR conditions: after an initial 10-min preincubation step (‘activation’ of the FastStart Taq DNA polymerase) at 95 °C, 60 amplification cycles were performed each consisting of 95 °C for 15 s, and 60 °C for 60 s. The fluorescent signals were measured after each primer-annealing step (60 °C).

The real-time PCR analysis was a relative quantification, with HER2 relative to a reference gene (Gastrin) performed in the same reaction well, relying on the assumption that the copy number of HER2 and the reference gene (Gastrin) is similar in healthy cells. Thus, the relative amount of HER2 can be calculated based on quantitative measurements in the real-time PCR. For data analysis, the PCR efficiencies (E) for HER2 and Gas were estimated to be very close to 2 (data not shown), and the calculation of the relative number (Ratio R) of HER2 and gastrin gene copy numbers was calculated as the mean of the calculated HER2/Gastrin ratios for each sample:


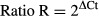


The threshold cycle (Ct) value is the real-time PCR cycle where the fluorescence signal of the logarithmic qPCR reaction crosses the threshold value, i.e., the PCR cycle where the fluorescence signal is above background noise [17]. The ΔCt value is the difference in Ct value between the reference gene (Gastrin) and HER2. Both reactions occur in the same reaction well with the final result being a quantification of HER2 relative to the reference gene (Gastrin). A ratio of 2.0 was considered HER2 overexpression. Ct values above 45 were not included in data analysis. Calculations and statistical analysis were performed on Excell software version 2003 (Microsoft, Redmont, WA, USA). Coefficient of variance (CV%) is calculated as mean value of replicates divided by standard deviation.

## Results

All 40 samples were analyzed for IHC, with two samples borderline positive for HER2. However, neither of the two IHC borderline positive samples was positive in FISH or real-time PCR (Tables 1 and 2). Concordance between IHC and FISH was 89.7% (35 of 39 samples), with two IHC positive samples negative in FISH and two IHC negative samples positive in FISH (Tables 1 and 2). Concordance between IHC and real-time PCR was 72.7%.

**Table 2:** HER2 amplification measured by IHC by two observers, FISH, and real-time PCR on laser microdissected cancer cells from ovarian carcinoma

Case no.	Stage	Tumor type	IHC	FISH HER2/Chr 17	Ratio DNA HER2/Gastrin N = 1–4
1	I	Endometrioid	0	1.11	1.48 [0.12–3.48] N = 3^1^
2	I	Endometrioid	0	1.20	0.63 [0.62–0.64] N = 2
3	I	Papillary serous	1	3.14	2.84 [0.47–5.20] N = 2^1^
4	I	Clear cell	0	1.03	0.33 [–] N = 1
5	I	Mucinous	0	1.07	0.93 [0.66–1.36] N = 4
6	I	Endometrioid	0	1.19	0.61 [0.42–0.80] N = 2
7	I	Mucinous	0	1.22	0.20 [0.16–0.35] N = 3
8	I	Endometrioid	1	1.23	4.06 [0.94–7.40] N = 3^1^
9	I	Papillary mucinous	0	1.43	0.49 [0.40–0.57] N = 3
10	I	Endometrioid	0	1.09	0.23 [0.10–0.53] N = 3
11	I	Mucinous	0	1.11	0.98 [0.47–1.68] N = 3
12	I	Clear cell + serous	2	1.65	0.75 [0.44–1.14] N = 3
13	I	Clear cell	0	1.74	0.23 [0.18–0.29] N = 3
14	I	Serous	0	1.11	Not Det.
15	I	Clear cell	1	1.32	Not Det.
16	II	Serous	0	1.10)	0.56 [0.02–1.08] N = 4
17	II	Clear cell + endometrioid	0	7: 1.71 & 8: 1.22	1.09 [0.18–2.65] N = 3^1^
18	II	Endometrioid	0	ND	0.92 [0.40–1.44] N = 2
19	III	Papillary serous	0	0.98	0.63 [0.30–0.75] N = 4
20	III	Papillary serous	1	1.13	8.10 [–] N = 1
21	III	Endometrioid	1	1.15	0.95 [0.53–1.36] N = 2
22	III	Clear cell	0	1.23	0.50 [0.07–1.28] N = 4
23	III	Serous	0	1.28	2.77 [0.90–6.81] N = 4^1^
24	III	Papillary serous	0	1.46	2.95 [0.11–5.78] N = 2^1^
25	III	Endometrioid	0	1.85	1.74 [1.36–2.48] N = 4^1^
26	III	Papillary serous	0	0.62	0.27 [–] N = 1
27	III	Endometrioid	0	0.98	1.14 [0.83–1.65] N = 3
28	III	Endometrioid	0	1.18	1.00 [0.94–1.11] N = 4
29	III	Serous + endometrioid	1	1.25	2.97 [0.65–8.19] N = 4^1^
30	III	Endometrioid	1	2.03	6.82 [0.94–16.33] N = 4^1^
31	III	Endometrioid	2	1.22	1.82 [1.61–2.03] N = 2^1^
32	III	Endometrioid	0	1.05	ND
33	III	Endometrioid	0	1.07	ND
34	III	Serous	0	1.2	ND
35	III	Serous	0	1.11	ND
36	IV	Serous	1	1.03	0.75 [–] N = 1
37	IV	Serous	0	1.17	0.78 [–] N = 1
38	IV	Clear cell + serous	1	1.28	3.51 [2.37–4.34] N = 2
39		Mucinous	1	1.15	6.21 [1.4–11.0] N = 2^1^
40	IV	Endometrioid	0	1.74	ND

FISH, fluorescence in situ hybridization; HER2, human epidermal growth factor receptor type 2; IHC, immunohistochemistry; ND, not determined; PCR, polymerase chain reaction.

1 The qPCR ratio between HER2 and gastrin has values both above and below cutoff.

**Fig. 2:**
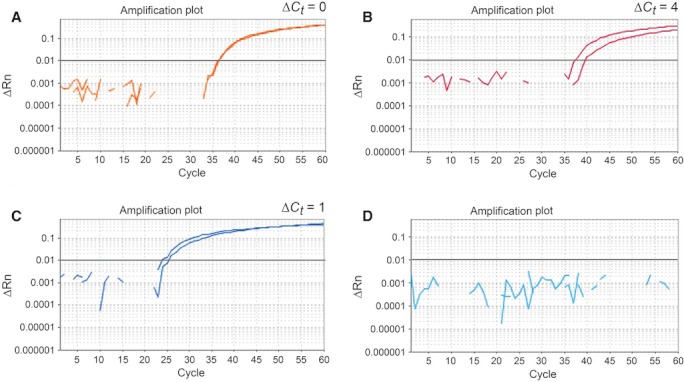
Amplification plots from the HER2 DNA copy-number real-time PCR assay. Representative plots depicting (A) non-amplification of HER2, (B) amplification of HER2, (C) DNA control, purified from blood, (D) H_2_O control, a blind sample to ensure the assay does not contain DNA contaminants.

Thirty-nine of the 40 samples (97.5%) were successfully analyzed for HER2 by the FISH method, with 37 HER2 negative (92.5%) and 2 HER2 positive (5.0%). Thirty-nine of the 40 samples measured by quadruple real-time PCR analysis showed real-time PCR reaction for either HER2 or Gastrin. Thirty-three samples (82.5%) had one or more relative measurements of HER2 DNA copy number, with mean Ct ranging from 34.0 to 42.0 (Table 3). Twenty-four (60.0%) of the samples analyzed by real-time PCR showed no HER2 DNA amplification (Tables 2 and 3, Fig. 2), whereas nine samples (22.5%) showed increased HER2 DNA copy number.

**Table 3:** Number of PCR reactions detected on microdissected ovarian carcinoma tissue

	No. of samples	CV%^1^	Ct range
No PCR reaction	1	–	–
PCR reaction in either HER2 or gastrin	6	–	38.3–43.7
1× PCR reaction in both HER2 and gastrin	5	–	37.0–40.0
2× PCR reaction in both HER2 and gastrin	9	68.3	36.6–42.0
3× PCR reaction in both HER2 and Gastrin	10	75.5	36.9–39.4
4× PCR reaction in both HER2 and gastrin	9	82.2	34.0–39.3

HER2, human epidermal growth factor receptor type 2; PCR, polymerase chain reaction.

1 Coefficient of variance (CV%) was calculated as the mean CV% of samples with either two, three, or four PCR reactions in both HER2 and gastrin.

Concordance between FISH HER2 analysis and real-time PCR from microdissected tissue was 78.1%, with 25 samples showing similar results in both FISH and real-time PCR, including the two FISH positive samples. Seven samples were dissimilar in FISH and real-time PCR, and in all cases, FISH was negative and real-time PCR was positive, with FISH results ranging from 1.13 to 1.46 and real-time PCR results ranging from 2.77 to 8.10 (Table 2). CV% was not measured on IHC or FISH; however, in the real-time PCR assay, the CV% was 68–82% (Table 3).

The positive samples were of the following type: IHC status showed two samples with suspicion of increased HER2, one clear cell + serous adenocarcinoma and one endometrioid adenocarcinoma. FISH analysis showed one serous and one endometrioid adenocarcinoma. qPCR analysis showed five serous, two endometrioid, one mucinous, and one clear cell carcinoma (Table 1).

## Discussion

In our study, we find that only 5.0% of cases were HER2 borderline positive (2+) by IHC analysis. In line with the IHC results, 5.0% of cases were HER2 amplified with the FISH method. In comparison, a study on tissue microarray from 27 ovarian cancer patients shows 14.8% HER2 positive result by FISH analysis [12]. Our result is in the low range of reports from ovarian cancer of 5–27% (11, 18–23). Currently, our data are the largest sample study on ovarian cancer using FISH in an unselected material with unbiased analysis of FISH in samples collected consecutively from 40 women. It has been reported that generally FISH analysis gives lower HER2 positive results than IHC analysis (18–23); however, in our material, IHC and FISH give similar percentage of positive results, although the two analyses do not agree which patients are positive. Some studies report that other receptors in the HER family are expressed in ovarian cancer and this opens the possibility that the activity of HER2 in ovarian cancer is more complex suggesting that formation of heterodimeric HER2/HERx receptors could facilitate HER2 oncogenic activity even in the absence of HER2 amplification (13, 24, 25). Our findings with a relatively low frequency of HER2 amplified ovarian cancers makes the concept of HER2 testing as a routine screening in ovarian cancer less obvious as testing of Trastuzumab adjuvant therapy in a clinical trial would require a very large cohort to give sufficient power [10]. However, HER2 diagnostics followed by therapy have improved the treatment of breast cancer patients and the concept of HER2 gene amplification leading to increased aggressiveness of the cancer is well documented (1–5). Thus, it remains an attractive idea to test existing and novel HER2 diagnostics and therapies in ovarian cancer, but the method of analysis should have a very high negative and positive predictive value to be included as a routine test in ovarian cancer.

In contrast to IHC and FISH results, the analysis of HER2 amplification by real-time PCR strongly increased the HER2 positive fraction from 5.0% (IHC and FISH) to 22.5%. This could imply that the IHC and FISH procedures underestimate HER2 status. IHC has long been viewed as a screening method, with borderline results (2+) verified by the gold standard method: FISH [15]. However, the use of up to 600 pure cancer cells (Fig. 1) in a microdissected sample in the real-time PCR analysis (Fig. 2) compared with 60 gene signals used in FISH ought to give a more accurate and reproducible result. A diagnostic test performed on the correct cells in a greater number should lead to a more accurate method [14]. However, the CV% on the real-time PCR analysis is 70–80%, and this influences the results exemplified by five samples having values on both sides of the cut-off value (>2). Moreover, the real-time PCR Ct values were very high (mostly over Ct 35) implying that either very little DNA was present in the purified sample or that the ovarian tissue contained PCR inhibitors. In either case, the real-time PCR analysis risks the ‘Monte Carlo’ effect, where small differences in PCR efficiency in early PCR cycles in reactions with little template give rise to large differences in the final real-time PCR analysis [17]. Analytical success was achieved in approximately 80% of samples with seven samples not giving results, and this calls for better robustness of the assay. CV% on FISH analysis was not performed in the current study, but literature shows a CV% of approximately 5% within lab and 12% between labs (26). Half (4 of 8) of the real-time PCR HER2 positive results come from cancers with the serous type histology; however, it is unclear whether this result is reflecting methodological issues or that the serous type tumors are more prone to HER2 amplification.

We expected that a HER2 assay performed on LCM ovarian cancer cells purified from all surrounding cells would greatly increase the reliability of the HER2 analysis compared with, e.g., the FISH analysis, where there is a risk of misinterpreting stromal and inflammatory cells as cancer cells. Also, we expected that an assay relying on objective quantitative measurements would outperform a FISH assay dependent on subjective interpretation. Therefore, we hoped that our real-time PCR DNA copy-number assay in the current study would show better analytical performance than FISH. In our hands, the in-house HER2 real-time PCR assay including DNA preparation would have to be optimized and the CV% significantly reduced before the assay could be recommended for clinical use and can so far only be used for research. However, the fact that HER2 is detected in more patients by the real-time PCR method together with the fact that some patients negative in HER2 by FISH in previous studies have had positive effects of trastuzumab imply that more accurate HER2 tests are needed. Further studies with improved preanalytical processing are warranted to evaluate the promising concept of LCM and real-time PCR analysis. Moreover, the real-time PCR assay should be tested in different tissues, e.g., breast or the GI tract where PCR inhibitors could be less abundant.

In conclusion, IHC/FISH analysis showed a small proportion of HER2 positive samples (5.0%) which is comparable with previously published results and supports reports of low HER2 overexpression in ovarian cancer. CV% values of the real-time PCR assay are too high to be applicable in a clinical setting, and the assay needs more robustness to be a routine method in assessment of HER2 status in ovarian cancer. Further studies are needed to confirm ovarian cancer as a tissue with low HER2 overexpression and to evaluate the promising concept of LCM and real-time PCR analysis.
